# The Chicago Journal of Nervous and Mental Diseases

**Published:** 1874-01-01

**Authors:** 


THE CHICAGO JOURNAL OF NERVOUS AND MENTAL DISEASES.—This is the title to a new medical periodical to be published in this city by Prof. Y. S. Jewell and Henry M. Bannister, M.D., to be devoted to the special department of nervous and mental diseases. The first number is promised about the first of February next, and is to be continued quarterly thereafter. We know that the editors and proprietors of the proposed journal have been making the most ample preparations for their work, and we hope they will be rewarded by a liberal patronage from the profession.

